# Miscellaneous

**Published:** 1889-05

**Authors:** 


### MISCELLANEOUS.

THE LAWN.—The artistic arrangement of the lawn is, to the owner of every country place, a matter of the first importance, or should be. Time was when flower beds plentifully besprinkled its green surface, and made of even the smallest plot a very garden of brilliant coloring, attracting the eye of every beholder. But the fashions change here as well as elsewhere, and behold, now, the popular taste would point toward the larger expanse of green with its modicum of shrub and tree, while the old time flower bed and its occupants smile at you through the garden fence.

Care should be taken to have a firm, beautiful sod in the first place, to which end a thorough preparation is essential, and, whether it is to be sodded or sown, frequent top-dressing will insure its continued beauty, especially if cut close and often.

As to the arrangement, a continuous variety in form, color and expression would lend themselves to the eye and hand of the true lover of artistic beauty. Here a certain species of tree, dainty and delicate in nature, yet perfectly hardy, would form a beautiful mass when grouped, like the dwarf Arbor Vitæ and Irish Juniper, while there are for separate planting the varieties of Norway Spruce, White Spruce and Hemlock, the larger varieties to be kept within bounds by cutting back, while the dwarfish kinds may follow their will.—*Vick's Magazine.*

SAGE-TEA, or rum and quinine, or tincture of cantharides rubbed into the scalp may stop hair from falling out.

In these days of food adulteration it is gratifying to find an article for the table that is thoroughly reliable. Walter Baker & Co.'s breakfast cocoa is eminent in this limited class. No chemicals are used in its manufacture, it is absolutely pure, and forms moreover a delicious and healthful drink, as refreshing, and more nutritious, than tea or coffee, and free from the injurious effects that those beverages sometimes produce. And it is very cheap withal. The house of Walter Baker & Co. has maintained for more than 100 years a great and honored repute by the excellence and purity of its manufactures.

A VICTIM of inebriety attributes his cure to the following prescription : Make a vinegar of ground quassia, a half ounce steeped in a pint of vinegar, and put about a teaspoonful of it in a little water, and drink it down every time the liquor thirst comes on violently. I found it satisfied the cravings and suffused a feeling of stimulation and strength. For two years I have not tasted liquor, and I have no desire for it. Lately to try my strength, I have handled and smelt whiskey, but I have no temptation to take it. I give this for the consideration of the unfortunate, several of whom I know have recovered by the same means which I no longer require to use.

THE LATENT COLOR OF MATERIAL.—In a recent communication to the Paris Academy of Sciences, M. G. Govi has brought forward some interesting facts as to the true colors of bodies. In general, if any substance is illumined by monochromatic light only, it will appear black, except when the color of the light is the same as that naturally transmitted or diffused by the material in question. Thus carmine is black when viewed by either green or blue light, ultramarine is black in all but blue light, and similarly with most other colors. Reasoning from this, it would naturally be concluded that bin-iodide of mercury and red lead would appear black, when seen by the sodium light ; which is almost a pure mono chrome, while M. Govi's experiments show that in place of darkening, their color becomes a brilliant yellow, losing all trace of its initial red. Again, if bin-iodide of mercury and vermilion, which in ordinary sunlight appear nearly identical, are seen in the sodium light, the bin-iodide becomes a pale yellow, while the vermilion appears brown. M. Govi attributes the above to the fact that the sodium rays are wanting in sunlight, and thus with it the true colors of these bodies do not appear.

AN EXTRAORDINARY BEARD.—Louis Coulon, a Frenchman, at the age of sixty-three years, is the possessor of a beard seven feet and nine inches in length. This unusual capillary development commenced at the early age of twelve years, when he was obliged to shave regularly, but the growth soon became unmanageable by the razor, and he allowed it to attain its present length. Coulon is a miller by trade, and has always refused to put himself and his beard on exhibition. There is no apparent reason for the remarkable activity displayed by his capillary glands and he can only be set down as a freak of nature, in contrast to those less fortunate persons upon whose head and face the natural covering refuses to grow at all. There are numerous traditions of persons living in former times with beards even exceeding in length that of Coulon, but they are not well authenticated.

EXPERIENCE has proved the CELLULOID COLLAR AND CUFF to be a prime necessity, especially in hot weather. They never wilt, are soft and pliable, and can be worn without change for months. They are the most economical goods worn—no expense for laundering. If soiled, simply wash in soap and water and they are ready again for use. ☞ A complete assortment of these goods is sold by GEO. CLEMENT & Co., 33 East 22d St., New York.

